# Evaluation of *LOXL1* gene polymorphisms in exfoliation syndrome and exfoliation glaucoma

**Published:** 2008-03-17

**Authors:** Jose A. Aragon-Martin, Robert Ritch, Jeffrey Liebmann, Colm O’Brien, Karima Blaaow, Franco Mercieca, Anthony Spiteri, Caroline J. Cobb, Karim F. Damji, Ahti Tarkkanen, Tayebeh Rezaie, Anne H. Child, Mansoor Sarfarazi

**Affiliations:** 1 Molecular Ophthalmic Genetics Laboratory, University of Connecticut Health Center, Farmington, CT; 2 Cardiac and Vascular Sciences, St. George’s University of London, London, United Kingdom; 3 Einhorn Clinical Research Center, New York Eye and Ear Infirmary, New York, NY; 4 Department of Ophthalmology, Mater Misericordiae University Hospital, Dublin, Ireland; 5 Department of Ophthalmology, St. Luke’s Hospital, Guardamangia, Malta; 6 Department of Ophthalmology, Ninewells Hospital, Dundee, Scotland; 7 University of Ottawa Eye Institute, Ottawa, ON, Canada; 8 Department of Ophthalmology, Helsinki University Eye Hospital, Helsinki, Finland

## Abstract

**Purpose:**

To evaluate genetic susceptibility of *lysyl oxidase-like 1* (*LOXL1*) gene polymorphisms to exfoliation syndrome (XFS) and exfoliation glaucoma (XFG) in a case-control cohort of American and European patients.

**Methods:**

DNA from a total of 620 individuals including 287 exfoliation patients and 333 healthy control subjects were extracted by standard methods. Three single nucleotide polymorphisms (SNPs) of rs1048661 (R141L), rs3825942 (G153D), and rs2165241 were genotyped in these individuals by SNaPshot Assay. The seven coding exons of the *LOXL1* gene and their immediate flanking regions were directly sequenced in 95 affected patients. Data management and case-control association studies were performed with SNP-STAT and PLINK programs. The obtained DNA sequences were evaluated with the STADEN package.

**Results:**

The 287 unrelated exfoliation cases comprised of 171 American patients (mostly of European background) and 116 patients from 12 European countries. This phenotype was further divided into patients with exfoliation only and no glaucoma (XFO; n=95), exfoliation with glaucoma (XFG; n=133), and exfoliation unclassified (XFU; n=59). Genotypic data were analyzed separately for XFO, XFG, XFU, and XFS (all exfoliations; n=287) and for Americans and Europeans. The observed genotypic frequencies for each exfoliation phenotype or population were tabulated separately and tested for deviation from the Hardy–Weinberg equilibrium (HWE) using a standard Χ^2^ test. There were no HWE deviations and no significant genotypic differences between these subcategories for the three studied SNPs. For the combined exfoliation cohort, homozygote genotypes of G/G (rs1048661), G/G (rs3825942), and T/T (rs2165241) were significantly overrepresented. Likewise, case-control allelic association for rs1048661 (p=7.74x10^−9^), rs3825942 (p=3.10x10^−17^), and rs2165241 (p=4.85x10^−24^) were highly significant. The corresponding two-locus haplotype frequencies of GG for rs1048661-rs3825942 (p=1.47x10^−27^), GT for rs1048661-rs2165241 (p=1.29x10^−24^), and GT for rs3825942-rs2165241 (p=2.02x10^−24^) were highly associated with exfoliation phenotypes. The combined effect of these three SNPs revealed that the GGT haplotype is overrepresented by 66% in exfoliation cases, and this deviation from controls is highly significant (p=1.93x10^−24^). This haplotype constituted a major risk factor for development of exfoliation in both XFS and XFG. By contrast, the GAC haplotype was significantly underrepresented (p=4.99x10^−18^) in exfoliation cases by 83% and may potentially have a protective effect for this condition with an estimated attributable risk percent reduction of 457%. The only other haplotype that was significantly different between cases and controls was TGC (p=5.82x10^−9^). No observation was made for the GAT haplotype. The combined three haplotypes of GGT, GAC, and TGC were associated with 91% of the exfoliation syndrome cases in the studied populations. Seven coding exons of *LOXL1* were also sequenced in 95 affected cases. In addition to the three above-mentioned SNPs, 12 other variations were also observed in these patients (G240G, D292D, A320A, V385V, rs2304719, IVS3+23C>T, IVS3–155G>A, IVS3–101G>A, IVS4+49G>A, rs2304721, IVS5–121C>T, and rs2304722). None were considered a disease-causing mutation.

**Conclusions:**

We confirmed a strong association with *LOXL1* variants in our patients. For the *LOXL1* gene, individual alleles of rs1048661 (G), rs3825942 (G), and rs2165241 (T) are highly associated with XFS and XFG in American and European populations. The GGT haplotype constitutes a major risk haplotype for exfoliation, and GAC may have a protective role. DNA sequencing of 95 affected patients did not show any mutations in this gene. The *LOXL1* SNPs are located in the 15q24.1 band and within a genetic locus (GLC1N) that is associated with primary open-angle glaucoma (POAG). However, the *LOXL1* genetic predisposition is only limited to exfoliation with or without glaucoma and does not include the POAG phenotype.

## Introduction

Exfoliation syndrome (XFS) is an age-related, generalized disorder of the extracellular matrix characterized by the production and progressive accumulation of a fibrillar extracellular material in many ocular tissues [1]. It is now understood to be the most common identifiable cause of open-angle glaucoma worldwide, accounting for the majority of cases of this disease in some countries [2]. Its incidence increases progressively with age while its widespread distribution, its frequency, and its potential association with other diseases is only beginning to be realized.

All anterior segment structures are involved in XFS. Deposits of white material on the anterior lens surface are the most consistent and important diagnostic feature. The classic pattern consists of three distinct zones that become visible when the pupil is fully dilated, a central disc, an intermediate clear zone created by the iris rubbing exfoliation material from the lens surface during its physiologic excursions, and a granular peripheral zone [1]. Exfoliation material is often found at the pupillary border.

Exfoliation material is a complex glycoprotein/proteoglycan structure bearing epitopes of the basement membrane and elastic fiber system. The characteristic fibrils, which are composed of microfibrillar subunits surrounded by an amorphous matrix comprising various glycoconjugates, contain predominantly epitopes of elastic fibers such as elastin, tropoelastin, amyloid P, vitronectin, and components of elastic microfibrils such as fibrillin-1, microfibril-associated glycoprotein-1, and latent transforming growth factor beta-binding proteins (LTBP1 and LTBP2) by immunohistochemistry [1,3].

The risk of developing glaucoma is 5–10 times more common in eyes with XFS than in those without it. About 25% of patients with XFS have elevated intraocular pressure (IOP), and one-third of these have glaucoma. Patients with XFS are twice as likely to convert from ocular hypertension to glaucoma, and when glaucoma is present, it progresses more rapidly [4-6].

Exfoliation syndrome leads not only to severe, chronic open-angle glaucoma but may also lead to lens subluxation, angle-closure, blood-aqueous barrier impairment, and serious complications at the time of cataract extraction such as zonular dialysis, capsular rupture, and vitreous loss. There is increasing evidence for an etiological association of XFS with cataract formation and with retinal vein occlusion. Deposits of exfoliation material have been found in the heart, lung, liver, kidney, gall bladder, and cerebral meninges by electron microscopy [7,8].

An increasing number of associations with specific systemic disorders, primarily related to vasculopathy, has been reported including transient ischemic attacks [9], hypertension, angina, myocardial infarction, stroke, asymptomatic myocardial dysfunction [10], Alzheimer disease [11-13], and hearing loss [14,15].

Exfoliation syndrome is seemingly inherited as an autosomal dominant condition as evidenced by the largest available pedigree described in Nova Scotia [16]. Although, mitochondrial and even multifactorial modes of inheritance have also been suggested for XFS [17], familial cosegregation has been observed in many populations. A genome-wide linkage study of the Finnish population has recently identified a promising genetic locus on 18q with a multipoint LOD score of 4.2 as well as other potential loci on 2q, 17p, and 19q [18]. We also identified a provisional locus on the 2q36 region, but mutation screening of over 20 genes has not as yet identified the defective molecule for XFS (unpublished data).

A recent genome-wide association study in the Icelandic population identified multiple single nucleotide polymorphisms (SNPs) in the *lysyl oxidase-like 1* (*LOXL1*) gene on 15q24.1 that are highly associated with the exfoliation phenotype [19]. Replication studies in the Swedish population confirmed genetic susceptibility of *LOXL1* polymorphisms to exfoliation with (XFG) or without glaucoma (XFS) [19]. However, no genetic association was observed in a group of unrelated primary open-angle glaucoma (POAG) for either of these two populations.

In this study, we investigated the role of *LOXL1* polymorphisms in 620 American and European patients (287 exfoliation and 333 controls). Our study confirmed that the *LOXL1* polymorphisms are highly associated with both XFS and XFG patients in the two populations studied.

## Methods

### Clinical diagnosis

The diagnosis of exfoliation syndrome was made by direct visualization on a slit-lamp examination of the typical pattern of exfoliation material on the anterior lens surface after pupillary dilation. Exfoliative glaucoma was diagnosed as those showing the characteristics of a history of intraocular pressure greater than or equal to 22 mmHg and a presence of typical glaucomatous optic disc cupping and visual field loss.

### Patient population

The patient population was composed of two major subgroups, Americans and Europeans (Table 1). A total of 171 American exfoliation cases (93 XFG) were examined and clinically diagnosed by two of the authors (R.R. and J.L.). All but five patients in this subgroup were of European ancestry.

**Table 1:** Distribution of exfoliation patients with no glaucoma (XFO), exfoliation with glaucoma (XFG), and exfoliation unclassified (XFU) in American and European patients.

**Subtype**	**American**	**European**	**Total**
XFO	72	23	95
XFG	93	40	133
XFU	6	53	59
XFS	171	116	287

XFS represents all exfoliation groups combined.

The remaining 116 patients (40 XFG) were from 12 European countries, primarily Irish, Scottish, English, Finnish, or Maltese. Their exfoliation and glaucoma diagnoses were made by their respective ophthalmologists. Several of them were found to have additional family members affected with exfoliation or glaucoma in a pattern suggesting autosomal dominant inheritance [17,20]. Many of these family members were clinically and genetically ascertained, and their DNA samples were collected. However, for this case-control association study, only one affected member per family was used.

Of the 287 unrelated exfoliation patients included in this study (Table 1), only 133 (93 Americans) were reported to have exfoliation glaucoma (XFG). Further, 95 (72 Americans) cases were reported to have only exfoliation (XFO) with no glaucoma. The glaucoma status for the remaining 59 (six Americans) unrelated patients was not reported, and thus, these cases were considered as unclassified (XFU). Altogether, 287 patients with exfoliation syndrome (XFS) including 133 XFG were used for statistical evaluation.

Similarly, we used a group of 333 unrelated healthy control subjects from the United States and Europe. To the best of our knowledge, none of the control individuals who participated in this study was related to our exfoliation patients. All of these healthy controls were clinically screened for the presence of exfoliation and glaucoma, and the majority of them declared as having a European genetic background. All the controls were between 60 and 98 years old and so they were age- and ethnically-matched with our exfoliation group.

The inclusion of human subjects in our study was approved by the University of Connecticut Health Center Institutional Review Board.

### SNaPshot genotyping assay

SNP genotyping was performed by the SNaPshot Assay. Unmodified, flanking oligonucleotide primers were synthesized and the polymerase chain reaction (PCR) product of each targeted SNP was generated separately by standard PCR reactions as described below. DNA samples from 620 unrelated exfoliation cases and controls were used for PCR amplification of three different *LOXL1* SNP markers, and their PCR products were pooled together separately for each individual. We also synthesized three other internal primers that would anneal to sequences adjacent to the exact site of each SNP marker. Subsequently, the pooled PCR products were subjected to a second round of PCR amplification using the ABI-SNaPshot Multiplex Kit and unlabeled internal primers. The new PCR reaction extended by only one nucleotide base at the exact site of the SNP and then terminated. The SNaPshot Multiplex Kit contains four ddNTPs that are fluorescently labeled with a different color dye. Since the length of our internally designed primers differed by at least 10 base pairs (bp), the newly generated fragments varied by size for various SNPs and by color for the allelic polymorphism within each SNP. Therefore, based on the two distinctive color and size differences, these multiplex PCR products were separated on an ABI-3100 Gene Analyzer instrument, and the resultant products were sized and genotyped by the ABI-GeneMapper Fragment Analysis Software (version 3.5).

#### SNaPshot PCR reaction

Each purified PCR product (1 μl) was added to 4 μl of a master mixture (0.5 μl SNaPshot mix, 0.2 μl of each SNaPshot primer [10 pmol/μl], and 3.3 μl of deionized H_2_O) and subjected to a second PCR reaction (35 cycles of 96 °C/10 s, 50 °C/5 s, and 60 °C/30 s). The SNaPshot products were purified by the shrimp alkaline phosphatase (SAP) treatment and then run on an ABI-3100 Gene Analyzer instrument. The genotyping of samples were performed with the help of GeneMapper software (version 3.5).

### Polymerase chain reaction

A set of primers was designed (forward: 5′-AAG GCC AGC ATG GAC AAA GCT AGA-3’ and reverse: 3’-GTA GTA CAC GAA ACC CTG GTC GTA GGT-5′) to amplify a 751 bp fragment from exon 1 of *LOXL1* that contained the two SNPs (rs1048661 [R141L] and rs3825942 [G153D]). A second primer set was designed (forward: 5′-TTC TTA GAA TGC AAG ACC TCA GC-3′ and reverse: 3′-CTC AGG GTA GTG GCC AGA GG-5′) to amplify a 269 bp fragment from intron 1 that carried the rs2165241 SNP. PCR reactions were performed to amplify a specific fragment of genomic DNA in the MJ Research-PTC-200 Peltier Thermal Cycler. The standard PCR program was performed under the following conditions: 96 °C/2 min, 55 cycles (96 °C/30 s, 63 °C/30 s, and 72 °C/30 s), and 72 °C/5 min final elongation. All the PCR products were checked for amplification by agarose gel electrophoresis and subsequently purified by SAP and Exo-I treatment.

### DNA Sequencing

A series of oligonucleotide primers were synthesized for amplification of seven known coding exons of *LOXL1*. Each set was designed with Primer3 software and covered at minimum 200 bp of the flanking intronic sequences (primers are available). Exon 1 was amplified and sequenced in two overlapping fragments. Each exon was amplified separately; their PCR products were purified, directly sequenced with BigDye Terminator (version 3.1) Cycle Sequencing Kit, and run on an ABI PRISM 3100 DNA sequencing instrument. The data were transformed to the STADEN package, and sequences were aligned together for each exon and for all of the 95 individuals used for this part of our study.

### Statistical analysis

The genotypic data as determined by the SNaPshot assay were imported into an in-house SNP data management program (SNP-STAT). The observed number of genotypes for each SNP were counted and the genotypic and allele frequencies were tabulated automatically. The two-locus genotypic counts and frequencies were also estimated for each set of SNP pairs. The standard Χ^2^ was used to test for deviation from the Hardy–Weinberg equilibrium and to compare the genotypic and allelic frequencies in exfoliation and control groups. Odds ratios, attributable risk percents (AR%), population attributable risk percents (PAR%), and p-values were calculated, and their appropriate confidence intervals were computed at the 95% level. For each sub-phenotype (XFO, XFU, XFG, and XFS) and for each population (American and European), this process was repeated. The SNP-STAT program was further used to export SNP information together with the entire genotypic data for use with the PLINK program [21]. All the statistical analyses were re-evaluated by the PLINK, and in addition, this program was used to determine the most likely haplotype-phases for the *LOXL1* SNPs and to further estimate their frequencies in exfoliation and control groups.

## Results

All of 287 cases used in this study had XFS (Table 1). Subsets had no glaucoma (XFO, n=95), glaucoma (XFG, n=133), or remained unclassified (XFU, n=59). For each of these phenotypic subgroups and for each of the three SNPs of the *LOXL1* gene, we tabulated both genotypic and allelic counts separately (Table 2 and Table 3) and made a cross-comparison statistical analysis by using a standard Χ^2^ test. Table 2 shows allelic counts and corresponding uncorrected p-values for each of the three SNPs. Of the 18 multiple tests shown in this table, only the p-value between XFO and XFG was significantly different. However, if one uses the Bonferroni correction method that multiplies uncorrected p-values by the number of comparisons performed, the reported p-value in Table 2 becomes almost insignificant, and such marginal p-values are always expected when a large number of statistical comparisons are made. Table 3 presents a detailed account of all genotypic and allelic counts and frequencies that were observed for each of these clinical subtypes and the three studied SNPs. Interestingly, when each of these exfoliation subtypes was compared with the normal controls, a significant association was detected with all the three *LOXL1* SNPs. Therefore, as no major allelic differences were observed between these phenotypic subgroups and as each subtype is highly associated with the three SNPs, we combined them as a single phenotype (exfoliation syndrome, XFS) for subsequent statistical evaluations. The observed genotypic frequencies in Table 3 were tested for possible deviations from the Hardy–Weinberg Equilibrium (HWE) in both the exfoliation and control groups. No deviation was observed from the HWE expectation for any of these two groups. As shown in Table 3, all three SNPs are highly associated with every single one of these clinical subtypes. For the two coding SNPs, rs1048661 (R141L) and rs3825942 (G153D), the two genotypes of G/G (p=2.85x10^−8^ and p=7.44x10^−15^) and the two corresponding alleles of G (p=7.74x10^−9^ and p=3.10x10^−17^) are highly overrepresented in the exfoliation cases (XFS) as compared to the control group. Likewise, for the intronic SNP of rs2165241, genotype T/T (p=1.24x10^−22^) and its corresponding allele T (p=4.85x10^−24^) are highly associated with XFS. Both the genotypic and allelic case-control association tests for each of these three SNPs and for each clinical subtype were highly significant (Table 3). We also performed additional statistical evaluations, tabulated odds ratios (OR), and population attributable risk percentages (PAR%) for each of these three SNPs and under various inherited genetic parameters (Table 4). The allelic PAR% values for rs1048661, rs3825942, and rs2165241 were estimated as 28%, 58%, and 32%, respectively (Table 4). These PAR% values represent theoretical estimates for the excessive rate of XFS in the American and European populations that are due to risk-associated factors in *LOXL1* polymorphisms. Therefore, if these specific SNP-associated risk factors are eliminated, the incidence of XFS in the population is expected to reduce by these percentages per each SNP.

**Table 2:** Comparison of allelic counts and the corresponding p-values for *LOXL1* SNP markers of rs1048661,rs3825942, and rs2165241 and different subtypes of exfoliation syndrome.

**Exfoliation subtype**	**Name of SNP**	**Allele types**	**Allele counts**	**Exfoliation subtype**		
				**XFG**	**XFU**	**XFS**
**XFO** (n=95)	rs1048661	G/T	156/32	p1=0.517*	p1=0.780	p1=0.675
	rs3825942	G/A	173/15	p2=0.001	p2=0.121	p2=0.034
	rs2165241	C/T	51/135	p3=0.504	p3=0.672	p3=0.823
**XFG** (n=133)	rs1048661	G/T	225/39	-	p1=0.800	p1=0.724
	rs3825942	G/A	260/4	-	p2=0.216	p2=0.054
	rs2165241	C/T	65/199	-	p3=0.301	p3=0.548
**XFU** (n=59)	rs1048661	G/T	96/18	-	-	p1=0.986
	rs3825942	G/A	110/4	-	-	p2=0.782
	rs2165241	C/T	35/83	-	-	p3=0.494
**XFS** (n=287)	rs1048661	G/T	477/89	-	-	-
	rs3825942	G/A	543/23	-	-	-
	rs2165241	C/T	151/417	-	-	-

* p1, p2 and p3 are the corresponding p-values for the three *LOXL1* SNPs as listed for each exfoliation subtype. These are uncorrected allelic p-values when two set of exfoliation subtypes are compared together.

**Table 3:** Genotypic and allelic counts, frequencies, and p-values for three *LOXL1* polymorphisms in different subtypes of exfoliation syndrome.

**Phenotype**	***LOXL1* SNPs**	rs1048661 **(R141L)**		rs3825942 **(G153D)**		rs2165241	
		**GG/GT/TT**	**G/T**	**GG/GA/AA**	**G/A**	**CC/CT/TT**	**C/T**
XFO (n=95)	Counts	62/32/0	156/32	79/15/0	173/15	4/43/46	51/135
	Frequency	0.660/0.340/0.0	0.830/0.170	0.840/0.160/0.0	0.920/0.080	0.043/0.462/0.495	0.274/0.726
	p-Value	1.37x10^-3^	5.44x10^-4^	9.18x10^-4^	1.01x10^-4^	5.99x10^-11^	2.30x10^−11^

XFG (n=133)	Counts	95/35/2	225/39	128/4/0	260/4	9/47/76	65/199
	Frequency	0.720/0.265/0.015	0.852/0.148	0.970/0.030/0.0	0.985/0.015	0.068/0.356/0.576	0.246/0.754
	p-Value	1.49x10^-5^	2.53x10^-6^	1.21x10^-11^	5.59x10^-13^	4.83x10^−17^	4.17x10^-17^

XFU (n=59)	Counts	40/16/1	96/18	53/4/0	110/4	3/29/27	35/83
	Frequency	0.702/0.280/0.018	0.842/0.158	0.930/0.070/0.0	0.965/0.035	0.051/0.491/0.458	0.297/0.703
	p-Value	8.39x10^-3^	2.17x10^-3^	1.25x10^-4^	1.67x10^-5^	8.24x10^-7^	3.28x10^-7^

XFS (n=287)	Counts	197/83/3	477/89	260/23/0	543/23	16/119/149	151/417
	Frequency	0.696/0.293/0.011	0.843/0.157	0.919/0.081/0.0	0.959/0.041	0.056/0.419/0.525	0.266/0.734
	p-Value	2.85x10^-8^	7.74x10^-9^	7.44x10^-15^	3.10x10^-17^	1.24x10^-22^	4.85x10^-24^

Controls (n=333)	Counts	162/140/28	464/196	216/98/18	530/134	94/174/60	362/294
	Frequency	0.491/0.424/0.085	0.703/0.297	0.651/0.295/0.054	0.798/0.202	0.287/0.530/0.183	0.552/0.448

Genotypic and allelic p-values were tabulated individually between each of the exfoliation subtypes and controls (n=333).

**Table 4:** Association tests, odds ratios, and population attributable risk percent (PAR%) for three SNPs of the *LOXL1* gene.

**Genetic test**	rs1048661 **(R141L)**			rs3825942 **(G153D)**			rs2165241		
	**p-Value**	**Odds**	**PAR %**	**p-Value**	**Odds**	**PAR %**	**p-Value**	**Odds**	**PAR %**
Homozygote	1.00x10^-6^	0.088	7	5.00x10^-5^	0.001	4	5.39X10^-22^	0.069	38
Heterozygote	3.4x10^−5^	0.488	14	2.46x10^-12^	0.195	15	1.11X10^-11^	0.275	34
Dominant	2.72x10^-7^	0.421	19	2.31x10^-15^	0.165	19	6.09x10^-19^	0.203	54
Recessive	2.89x10^-5^	0.116	4	7.02x10^-5^	0.001	3	1.38x10^-13^	0.149	15
Alleles	7.74x10^-9^	0.442	28	3.10x10^-17^	0.168	58	4.85x10^-24^	0.294	32

Note that p-values provided under each of these three SNPs were obtained by comparing only the rare genotypes or alleles against other genotypes or alleles, respectively.

### *LOXL1* risk-associated haplotypes in exfoliation syndrome

To determine the combined effect of these polymorphisms on XFS, we also performed a series of statistical analyses for all possible haplotypes of the three SNPs. Table 5 summarizes the estimated frequencies of each haplotype and provides results of association tests between XFS and controls for each haplotype. For the first two SNPs (rs1048661, rs3825942), haplotype GG was overrepresented by 59% in the XFS cases (0.8021) as compared to controls (0.5030). This deviation was statistically significant (p=1.47x10^−27^). In contrast, the other two haplotypes of TG and GA were significantly underrepresented in the XFS cases (Table 5). The TA haplotype was not observed in the control samples. Comparison of the two haplotypes of GG and TG relative to GA had odds ratios of 7.87 (p=1.31x10^−22^) and 2.62 (p=1.51x10^−4^), respectively. The two haplotypes of GG and TG accounted for 96% of the XFS cases, and the GA haplotype had the lowest estimated risk with an attributable risk percent (AR%) value of 394.

**Table 5:** Estimated two- and three-loci haplotype frequencies for three *LOXL1* SNPs and their corresponding association tests between exfoliation syndrome and controls.

**SNP1 alleles**	**SNP2 alleles**	**XFS**	**Controls**	**Association tests between exfoliation and controls**			
rs1048661	rs3825942	**n=566**	**n=658**	**p-Values**	**Odds (95% C.I.)**	**AR%**	**PAR%**
G	G	0.8021	0.503	1.47x10^−27^	4.00 (3.10–5.18)	37	22
T	G	0.1572	0.2964	9.01x10^−9^	0.44 (0.33–0.59)	88	28
G	A	0.0406	0.2006	4.83x10^−17^	0.17 (0.11–0.27)	394	58

rs1048661	rs2165241	**n=560**	**n=650**	**p-Values**	**Odds (95% C.I.)**	**AR%**	**PAR%**
G	T	0.7284	0.4362	1.29x10^−24^	3.46 (2.71–4.41)	40	24
T	C	0.1499	0.287	1.14x10^−8^	0.44 (0.33–0.58)	91	28
G	C	0.1126	0.2653	2.26x10^−11^	0.35 (0.26–0.48)	135	36
T	T	0.0091	0.0115	0.6812	0.83 (0.26–2.62)	21	9

rs3825942	rs2165241	**n=560**	**n=654**	**p-Values**	**Odds (95% C.I.)**	**AR%**	**PAR%**
G	T	0.7364	0.4458	2.02x10^−24^	3.45 (2.71–4.40)	39	23
G	C	0.2266	0.3547	1.16x10^−6^	0.53 (0.41–0.69)	56	20
A	C	0.037	0.1995	1.49x10^−17^	0.16 (0.10–0.25)	430	60

Combined haplotypes*		**n=566**	**n=666**	**p-Values**	**Odds (95% C.I.)**	**AR%**	**PAR%**
GGT		0.7278	0.4382	1.93x10^−24^	3.43 (2.69–4.36)	40	23
GAC		0.0346	0.1973	4.99x10^−18^	0.15 (0.09–0.24)	457	60
TGC		0.1486	0.2877	5.82x10^−9^	0.43 (0.32–0.57)	94	29
GGC		0.0795	0.0661	0.3693	1.22 (0.79–1.88)	17	9
TGT		0.0097	0.0107	0.8595	0.84 (0.26–2.66)	19	8

Individual p-values for each haplotype, odds ratios, 95% confidence intervals, their associated attributable risks percentages (AR%), and population attributable risks percentages (PAR%) between exfoliation syndrome and controls are provided. * Order of the alleles are: rs1048661, rs3825942, and rs2165241.

For the two SNPs, rs1048661 and rs2165241, all four possible haplotypes were estimated in both cases and controls. For this pair, only the GT haplotype was prevalent in the XFS cases (0.7284 versus 0.4362), and this deviation (67%) from the control group was highly significant (p=1.29x10^−24^). The two haplotypes of TC (p=1.14x10^−8^) and GC (p=2.26x10^−11^) were statistically underrepresented in the XFS cases while the TT haplotype did not show any difference (p=0.6812) between the two groups (Table 5). The three haplotypes of GT, TC, and GC significantly accounted for 99% of the XFS cases. However, when the GT, TC, and GC haplotypes were compared relative to TT, the tabulated odds ratios of 2.01 (p=0.228), 0.629 (p=0.436), and 0.513 (p=0.261), respectively, were not significant. The GC haplotype had the highest AR% value of 135.

Likewise, for the last two pairs (rs3825942, rs2165241), only the GT haplotype was overrepresented in the XFS cases (p=2.02x10^−24^). The two haplotypes of GC and AC were significantly underrepresented in the cases. Comparison of the two common haplotypes of GT and GC relative to AC produced odds ratios of 8.74 (p=2.51x10^−23^) and 3.39 (p=1.08x10^−6^), respectively, and these two haplotypes accounted for 96% of XFS cases. In summary, cross comparisons between each two pairs of SNPs revealed that the GG, GT, and GT haplotypes were significantly overrepresented in the XFS patients. Seven of the other eight haplotypes were significantly more frequent in the controls.

When we tabulated the combined effect of these three SNPs on XFS, the GGT haplotype was significantly overrepresented (p=1.93x10^−24^) while the two other haplotypes of GAC and TGC were significantly underrepresented (Table 5). There were no significant differences between cases and controls for the two haplotypes of GGC and TGT. The GAT haplotype was not observed. The combined three haplotypes of GGT, GAC, and TGC were associated with 91% of the XFS in the study population. When the three haplotypes of GGT, GAC, and TGC were compared individually to non-associated haplotypes of GGC or TGT, the estimated odds ratios of 1.38 (p=0.152) or 1.98 (p=0.240), 0.15 (p=3.31x10^−10^) or 4.68 (p=8.55x10^−3^), and 2.34 (p=5.52x10^−4^) or 1.63 (p=0.410), respectively, were obtained. When the same three associated haplotypes were individually compared to the combined haplotypes of GGC or TGT, the estimated odds ratios of 1.44 (p=0.087), 6.42 (p=3.02x10^−10^), and 2.24 (p=6.13x10^−4^) were obtained, respectively. Interestingly, the lowest significant risk was associated with the GAC haplotype (reduced by 83%), which accounted for the greatest protection against XFS with an AR% associated value of 457 (Table 5).

In addition to the abovementioned case-control association studies, we also tabulated the odds ratios, AR%, and population attributable risk percent (PAR%) for each of these haplotypes. As presented in Table 5, risk reduction (or risk protection for developing XFS) for highly associated haplotypes of GA (SNPs 1 and 2), GC (SNPs 1 and 3), AC (SNPs 2 and 3), and GAC were 394, 135, 430, and 457, respectively. Once the combined effect of these three SNPs was considered collectively, this data suggests that the GGT haplotype is overrepresented by 66% in the affected patients and therefore, is a major risk factor for XFS. On the contrary, the GAC haplotype is underrepresented in the cases by 83% and therefore, may play a protective role against the development of XFS. Table 5 also provides the odds ratios and the 95% confidence intervals for each haplotype as well as percentages of theoretical reduction in overall incidence of exfoliation (PAR%) if the corresponding associated haplotype is to be eliminated (or elevated) from (or in) the population.

### DNA sequencing of *LOXL1* in exfoliation syndrome

To determine the potential effect of *LOXL1* mutations in XFS patients from the population under our study, we directly sequenced the seven coding exons of this gene in a total of 95 unselected and unrelated affected patients. The results of these sequencings are presented in Table 6. A total of 14 DNA variations were observed in this gene of which six were in the coding exons and eight were in the adjacent introns. In addition to R141L (rs1048661) and G153D (rs3825942) that were also used in our association studies, we identified two novel variations, G240G and V385V, each in 1 out of 95 patients. No other significant differences were observed from the normal referenced sequence.

**Table 6:** DNA sequencing results of 95 patients with exfoliation syndrome.

**Location**	**Nucleotide change**	**Amino acid change**	**SNP number**	**Wildtype homozygous**	**Number of subjects observed with this variant**	
					**Heterozygous**	**Homozygous**
**Exon 1**	CGG>CTG	R141L	rs1048661	61	33	1
	GGC>GAC	G153D	rs3825942	90	5	0
	GGC>GGT	G240G	-	94	1	0
	GAC>GAT	D292D	-	93	2	0
	GCG>GCT	A320A	-	82	12	1
**Exon 2**	GTG>GTC	V385V	-	94	1	0
**Intron 2**	IVS2+197C>T	-	rs2304719	84	11	0
**Intron 3**	IVS3+23C>T	-	-	94	1	0
	IVS3–155G>A	-	-	82	13	0
	IVS3–101G>A	-	-	66	28	0
**Intron 4**	IVS4+49G>A	-	-	94	1	0
**Intron 5**	IVS5+111C>A	-	rs2304721	79	15	1
	IVS5–121C>T	-	-	88	6	0
	IVS5–51T>C	-	rs2304722	67	28	0

A total of 14 DNA variations were observed in this gene of which six were in the coding exons and eight were in the adjacent introns.

## Discussion

Recent genome-wide association studies in the Icelandic population identified multiple SNPs from the *lysyl oxidase-like 1* (*LOXL1*) gene that were highly associated with XFS and XFG [19]. The same observations were also made in the Swedish population [19] and have further been confirmed for two other populations [22,23]. This topic has also been subjected to several commentaries and reviews [24-28].

We studied 620 subjects, 287 exfoliation and 333 healthy controls from American and European populations. All patients were genotyped using the SNaPshot Assay for three SNPs from *LOXL1* that were reported to have strong associations with XFS in the Icelandic and Swedish populations [19]. We also confirmed a strong association with *LOXL1* variants in our patients (Table 3–Table 5). The G alleles of rs1048661 (SNP 1) and rs3825942 (SNP 2) together with the T allele of rs2165241 (SNP 3) are highly associated with XFS and XFG. When two-locus haplotypes were tabulated between SNP 1 and SNP 2, the GG haplotype was overrepresented in the affected cases while the TG and GA haplotypes were significantly underrepresented in the cases. Comparison of the two haplotypes of GG and TG relative to GA had odds ratios of 7.87 (p=1.31x10^−22^) and 2.62 (p=1.51x10^−4^), respectively. The two haplotypes of GG and TG accounted for 96% of the XFS cases, and this observation is in full agreement with the original report for the Icelandic and Swedish populations [19]. Similarly, for each two-set of SNP, the GG, GT, and GT haplotypes were significantly overrepresented in the XFS patients. Seven of the other eight haplotypes were significantly more frequent in the control patients. When the combined effects of these three SNPs were tabulated, the GGT haplotype was significantly overrepresented while the two haplotypes of GAC and TGC were significantly underrepresented in the XFS patients (Table 5). These three haplotypes accounted for 91% of the XFS cases in the studied population. In summary, for these three SNPs, the GGT haplotype was overrepresented by 66% and constituted a major risk haplotype for XFS while the GAC haplotype was underrepresented by 83% and had the lowest associated risk in XFS patients. DNA sequencing of 95 affected patients did not show any mutations in *LOXL1* in our studied population.

LOXL1 belongs to a family of extracellular copper-requiring enzymes (i.e., LOX, LOXL1–4) that facilitate cross-linking of collagens and elastins through oxidative deamination of lysine or hydroxylysine side chains [29]. The reported *LOXL1* risk-associated polymorphisms in XFS [19] may be a significant finding as this condition is considered to be a type of elastosis that affect elastic microfibrils. However, as the most highly XFS-associated haplotypes were also present in 44%–50% of our control subjects (Table 5), it is not clear at this point how these naturally occurring variations work individually or cooperatively to contribute to this phenotype. Since LOXL1 interacts with other proteins [30,31] such as fibulin-5 (FBLN5) and elastin (ELN), it is likely that through these protein–protein interactions and their anticipated common biochemical pathways, the effect of such polymorphisms on XFS becomes more significant. The two highly associated SNPs of rs1048661 (R141L; basic arginine replaced by neutral and hydrophobic leucine) and rs3825942 (G153D; neutral and polar glycine replace by acidic aspartic acid) are part of the coding region of the LOXL1 protein, and these two amino acids are highly conserved during evolution. There is also a strong linkage disequilibrium between these two SNPs (D’=0.996) thus suggesting that the effect of these two amino acid polymorphisms on XFS is probably influenced by protein–protein interaction of LOXL1 with FBLN5, ELN, or other, unidentified LOXL1-interacting proteins. Identification of specific polymorphisms in *LOXL1* that are highly associated with XFS and XFG provide a good starting point for future research into the etiology of this condition. Although *LOXL1* null mice [32] have not been specifically reported to have any ocular phenotype resembling XFS, perhaps the study of such animals at very old stages of life and/or their cross-breeding with other animals lacking LOXL1-interacting proteins such as FBLN5 and ELN are now warranted. However, as *LOXL1* polymorphisms are frequently seen in normal patients, it is not clear at this point how such information can provide any immediate assistance to patients having this condition or being at high risk for development of XFS or XFG.

It is now possible to determine specific haplotype composition of *LOXL1* polymorphisms in an individual patient and use the anticipated population-related risk frequencies to categorize a person into a relatively high or low risk group. However, it is not clear at this point if such information should be used to alter the normal clinical management of an individual as these risk estimates are relative, tentative, conditional, and probably population specific. More importantly, such polymorphisms are also significantly observed in healthy control subjects. Therefore, it is unlikely that such information will be useful for immediate day-to-day clinical management of patients.

It is also interesting that *LOXL1* polymorphisms are highly associated with both XFS and XFG, but no such association was reported for subjects only affected with primary open-angle glaucoma [19]. This in turn suggests that other factors must exist that predisposes an individual to develop glaucoma. Therefore, it is likely that XFG represents a group of patients that were hereditarily predisposed to glaucoma, but they instead developed XFG either because of *LOXL1* associated polymorphisms impacting the predisposed glaucoma gene expression and protein function or because of other as yet unknown systemic, hereditary, or environmental factors. Further research into the role of the LOXL1 protein in the etiology of exfoliation syndrome and exfoliation glaucoma is urgently needed.
